# Heteromeric TRP Channels in Lung Inflammation

**DOI:** 10.3390/cells10071654

**Published:** 2021-07-01

**Authors:** Meryam Zergane, Wolfgang M. Kuebler, Laura Michalick

**Affiliations:** 1 Institute of Physiology, Charité—Universitätsmedizin Berlin, Corporate Member of Freie Universität Berlin, Humboldt-Universität zu Berlin, and Berlin Institute of Health, 10117 Berlin, Germany; meryam.zergane@charite.de (M.Z.); laura.michalick@charite.de (L.M.); 2 German Centre for Cardiovascular Research (DZHK), 10785 Berlin, Germany; 3 German Center for Lung Research (DZL), 35392 Gießen, Germany; 4 The Keenan Research Centre for Biomedical Science, St. Michael’s Hospital, Toronto, ON M5B 1W8, Canada; 5 Department of Surgery and Physiology, University of Toronto, Toronto, ON M5S 1A8, Canada

**Keywords:** heteromeric TRP assemblies, pulmonary inflammation, endothelial permeability, TRPC3/6, TRPV1/4, TRPC1/4

## Abstract

Activation of Transient Receptor Potential (TRP) channels can disrupt endothelial barrier function, as their mediated Ca^2+^ influx activates the CaM (calmodulin)/MLCK (myosin light chain kinase)-signaling pathway, and thereby rearranges the cytoskeleton, increases endothelial permeability and thus can facilitate activation of inflammatory cells and formation of pulmonary edema. Interestingly, TRP channel subunits can build heterotetramers, whereas heteromeric TRPC1/4, TRPC3/6 and TRPV1/4 are expressed in the lung endothelium and could be targeted as a protective strategy to reduce endothelial permeability in pulmonary inflammation. An update on TRP heteromers and their role in lung inflammation will be provided with this review.

## 1. Introduction

Pulmonary microvascular endothelial cells are a key constituent of the blood air barrier that has to be extremely thin (<1 µm) to allow for rapid and efficient alveolo-capillary gas exchange. Integrity of this barrier is mandatory to prevent airborne pathogens from entering the blood circulation and causing systemic infections and to avoid fluids such as plasma or blood entering the alveolar space. Yet, over the past years a considerable body of work has demonstrated that activation of Transient Receptor Potential (TRP) channels can disrupt endothelial barrier function via a Ca^2+^-dependent increase of endothelial permeability, resulting in the formation of lung edema and an inflammatory response characterized by immune cell infiltration [1,2,3,4]. TRP channels form a family of transmembrane cation-permeable channels that can be categorized into six subfamilies: TRPA (ankyrin), TRPC (canonical), TRPM (melastatin), TRPML (mucolipin), TRPP (polycystin) and TRPV (vanilloid) [5]. Members of all six TRP channel subfamilies are expressed in the lung endothelium [6,7,8,9] where they can mediate Ca^2+^ influx and signaling as a key second messenger regulating cellular adaptation in various physiological and pathophysiological scenarios including endothelial permeability, vasodilation, angiogenesis, thrombosis, and inflammation [3,10,11,12]. For example, TRPV1 has been demonstrated to initiate inflammatory responses in pulmonary endothelial cells [13] while TRPV4-mediated Ca^2+^ influx can cause gap formation between endothelial cells, promoting infiltration of immune cells such as monocytes and polymorphonuclear leukocytes (PMNs) into the alveolar space, as well as pulmonary edema formation and lung injury [14]. The subcellular mechanisms that determine why Ca^2+^ entry via different Ca^2+^ channels result in differential cellular responses are so far poorly understood, but likely relate to highly localized signaling effects in cellular sub-compartments [15].

To build a functional ion channel, TRP subunits have to assemble as a tetrameric structure [16,17]. Different TRP subunit assemblies differ in their specificities, ion conductances and activation mechanisms. For instance, a TRP channel consisting of four TRPV4 subunits reacts to arachidonic acid [18] and can be activated by a phosphorylation in Ser824 [19]. Interestingly, studies have shown that TRP channel subunits not only form homomeric, but also heteromeric structures [20,21], and that such heteromeric assembly determines channel ion conductance, e.g., for Ca^2+^. The stoichiometry of TRP heteromers influences the Ca^2+^ influx and is therefore important to investigate. For example, it has been found that heteromeric TRPV5/6 would have a reduced opening probability for Ca^2+^, if more TRPV6 subunits built the TRPV5/6 channel [17]. As Ca^2+^ is a key intracellular second messenger initiating stress responses, cytoskeletal reorganization, and junctional disassembly [20], TRP heteromers and their distinct Ca^2+^ gating can be expected to play a critical role in the regulation of endothelial permeability. Thus, it was found that heteromeric TRPC1/4 and TRPC3/6 could increase endothelial permeability [1,22].

In this review, we present an update on the molecular mechanisms of TRP channel heteromerization for well-characterized (TRPC1/4, TRPC3/6) as well as newly emerging (TRPV1/4) TRP heteromeric assemblies in the pulmonary endothelium. All three heteromeric assemblies are expressed in the lung and of relevance in lung inflammation. Pulmonary endothelial cells can initiate and amplify inflammatory processes in various diseases or syndromes such as ARDS (acute respiratory distress syndrome) or COVID-19 (coronavirus disease 2019) [14,23,24]. In this context, Ca^2+^ as a second messenger plays a key role in activating inflammatory signaling pathways and is regulated, amongst others, by heteromeric TRP assemblies. Here, we will provide a comprehensive overview on the role of these assemblies in acute pulmonary vascular inflammation and discuss relevant methodological approaches and limitations for their analysis.

## 2. Methodological Approaches and Limitations in the Analysis of Heteromeric TRP Assemblies

Homo- and heteromeric TRP assemblies are analyzed with respect to their functional properties, their structure and their mechanisms of regulation. In general, TRP channel complexes are difficult to analyze as low expression levels of endogenous heteromeric assemblies or moderate TRP channel subunit interactions require very sensitive methods and cannot be detected by less sensitive but commonly used strategies such as co-immunoprecipitation (Co-IP) [25]. As a consequence, most studies addressing the heteromeric structure of TRP channels rely on overexpression systems in HEK293 cells as a versatile tool to construct, modify and test TRP heteromeric assemblies which may, however, result in artificial heteromeric channel formation due to high protein expression.

The initial proof of an interaction between TRP channel subunits can be demonstrated by using the Proximity Ligation Assay [26,27] but is commonly performed by Co-IP [28]. Pitfalls of Co-IP are false positive or negative results due to low protein purity and due to subunit disassembly as a result of plasma membrane treatment with harsh lysis or permeabilization buffers. Hence, Co-IP should be combined with a second, more sensitive method like the yeast two-hybrid (Y2H) technique or Förster resonance energy transfer (FRET). Y2H systems face some difficulties when used for transmembrane proteins but have been successfully modified to demonstrate a heteromeric Drosophila retinal-specific TRP and TRPL (like) assembly [29] into a membrane-based yeast two-hybrid system for protein interactions in the plasma membrane [30]. The most sensitive approach, however, to investigate TRP heteromers directly in living cells remains FRET, which has been used extensively for the study of heteromeric assemblies within the TRPV and TRPC subfamilies [28].

To examine which structural domains of TRP channel subunits contribute to the stabilization of TRP heteromers, loss of function mutations are commonly utilized, e.g., for the analysis of the heteromeric assemblies of TRPV5/6 [17] or TRPC3/6 [28]. For functional analyses of TRP channel subunit heteromerization, whole-cell patch-clamp recordings are able to identify modulations in cation conductances relative to the corresponding homomeric assemblies (e.g., for TRPV5/6 [17]).

In in silico analyses, interaction sites for protein interaction can be predicted by molecular docking [31]. Such analyses, however, require detailed structural input of putative heteromeric TRP subunits and their interaction sites. Thus far, such data are largely lacking due to the difficulties inherent in the assessment of the stoichiometric structure of a single channel by X-ray crystallography due to TRP channels’ flexibility and multi-domain topology [32]. This knowledge gap can be filled in part by analysis of single channel structure by cryo-electron microscopy [33]. Both X-ray crystallography and cryo-electron microscopy are two indispensable tools, generating valid input data for a broad range of in silico studies, uncovering stoichiometric changes and potential of modulating their properties by heteromerization.

Atomic force microscopy (AFM) provides a very sensitive method for determination of TRP heteromer’s stoichiometry, after TRP subunits were marked with a monoclonal antibody each, as shown for TRPP2/V4 with a 2:2 stoichiometry in HEK293 by Stewart and colleagues in 2010 [34]. In 2017, Single Channel Single Molecule Determination (SC-SMD) has been established successfully to investigate, for instance, the stoichiometry of heteromeric TRP channels by tagging TRP channel subunits in a heteromeric assembly with different fluorescent molecules which then can be detected by Total Internal Reflection Fluorescence (TIRF) microscopy. Likewise, combination of cross-linking TRP channel subunits with each other in a heteromeric assembly followed by mass spectrometry analysis [35] may present a versatile alternative for future analyses of physical interactions and stoichiometry in heteromeric TRP assemblies.

At present, FRET analyses and whole-cell patch-clamp recordings present the most commonly used and sensitive methods to probe for structural and functional interactions of heteromeric TRP assemblies. AFM, SC-SMD and cross-linking in combination with mass spectrometry present novel and promising strategies for future analyses of heteromeric TRP channel subunit assemblies with regard to their individual stoichiometry (an overview of methodological approaches is provided in Table 1).

## 3. Heteromeric TRP Channel Assemblies and Their Molecular Mechanisms

### 3.1. Introduction to TRP Channel Assemblies

**Figure 1:**
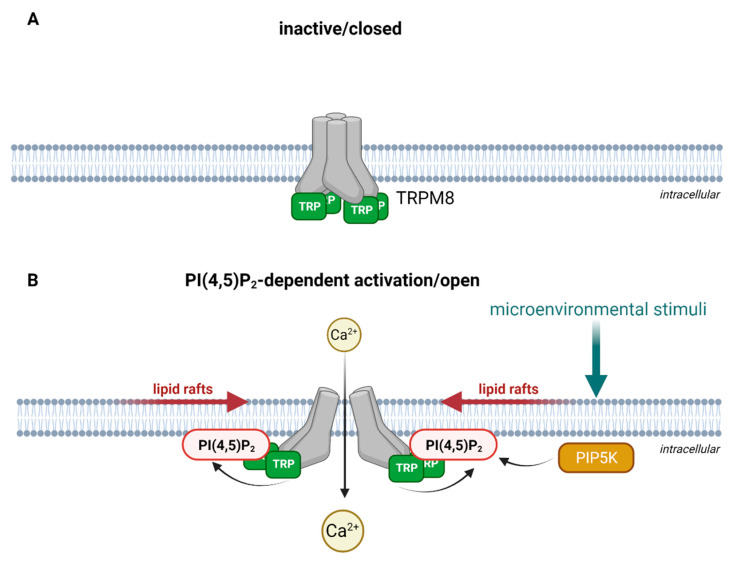
Schematic illustration of PI(4,5)P_2_-dependent activation as exemplified for TRPM8. (**A**) In the absence of PI(4,5)P_2_, the TRP-box holds the channel in a closed conformation. For simplification, other structural domains of TRPM8 are not illustrated. (**B**) Microenvironmental stimuli activate PI(4,5)P_2_ production via phosphorylation of phosphatidylinositol 4-phosphate by PIP5K (phosphatidylinositol-4-phosphate 5-kinase) in lipid rafts. The interaction of phosphatidylinositol 4,5-bisphosphate (PI(4,5)P_2_) the with the TRP-box activates TRPM8 and opens the channel for Ca^2+^ entry. Created with BioRender.com.

TRP channel subunits are membrane proteins, consisting of six transmembrane spans and a pore loop between the fifth and the sixth span that is cation-permeable [48]. The intracellular N- and C-termini are fitted with specific sequence motifs that are essential for the multimeric assembly of TRP channels. The N-terminus contains several ankyrin repeat domains (ARDs) [49], consisting of short sequences of 33 amino acid residues that form an anti-parallel helix-turn-helix structure followed by a β-hairpin loop. Several of these helix-turn-helix structures are packed into a bundle. These bundles form in turn a hand-shaped structure [50,51]. The C-terminus of TRPC, TRPV and TRPM subtypes contain a so-called TRP box, which maintains the channel in a closed confirmation in a phosphatidylinositol 4,5-bisphosphate (PI(4,5)P_2_)-dependent manner [52,53] as shown in Figure 1 upon the example of TRPM8.

Additionally, coiled-coil domains (CCDs) consisting of seven α-helices that interact with each other have been reported in TRP channels [54,55] with the potential to build coiled-coil structures [56]. CCDs have been also described to be differentially localized to C- and N-termini depending on the individual TRP subfamily and to promote multimeric assembly, channel maturation and trafficking [55]. For instance, the TRPM subfamily and TRPA1 contain CCDs on their C-terminus [57,58] whereas TRPC1 and TRPC4 have their CCDs on the N-terminus [59].

### 3.2. Heteromeric TRP Channel Assemblies

Heteromeric assemblies of TRP channel subunits are known to be formed within their subfamily and moreover with TRP channel subunits from other subfamilies, as it has been demonstrated for heteromeric assemblies of TRPP2/C1 in pig kidney epithelial cells [21], or TRPC1/V6 and TRPML/V5 in HEK293 cells [60,61]. Heteromerization of three different TRP channel subunits has also been described, e.g., for TRPC3/C6/C7 [62], TRPC1/C4/C5 [63], or TRPV4/C1/P2 [64] although heteromerization of two TRP channel subunits seems to be more commonly found [20]. It seems fair to speculate that with an increasing number of different TRP channel subunits being co-expressed in the same tissue or cell type, the probability for heteromerization increases which in turn may serve as a mechanism to fine-tune cellular responses to tissue-specific environmental stimuli [65]. For example, TRPV4, TRPC1 and TRPC6 have been shown to heteromerize in dorsal root ganglion neurons where they mediate mechanical hyperalgesia and primary afferent nociceptor sensitization [66] whereas TRPV4, TRPC1 and TRPC6 alone have no influence on the baseline mechanical nociceptive threshold [66,67,68]. Other examples for processes specifically regulated by heteromeric TRP channels include TRPC1/4 which mediates a large depolarizing plateau potential in lateral septal neurons that is the cause for epileptiform burst firing [69], TRPV1/4 induced angiogenesis in retinal microvascular endothelial cells (RMECs) in contrast to TRPV4 alone which inhibits angiogenesis in endothelial cells from prostate cancer [70], or TRPC3/6 which plays an important role in T-lymphocyte apoptosis and is increased in septic rat peripheral blood T-lymphocytes [71], indicating that heteromerization may also occur as an adaptive response to an inflammatory milieu.

### 3.3. Molecular Mechanisms for Stabilization of Heteromeric TRP Channel Assemblies

**Figure 2:**
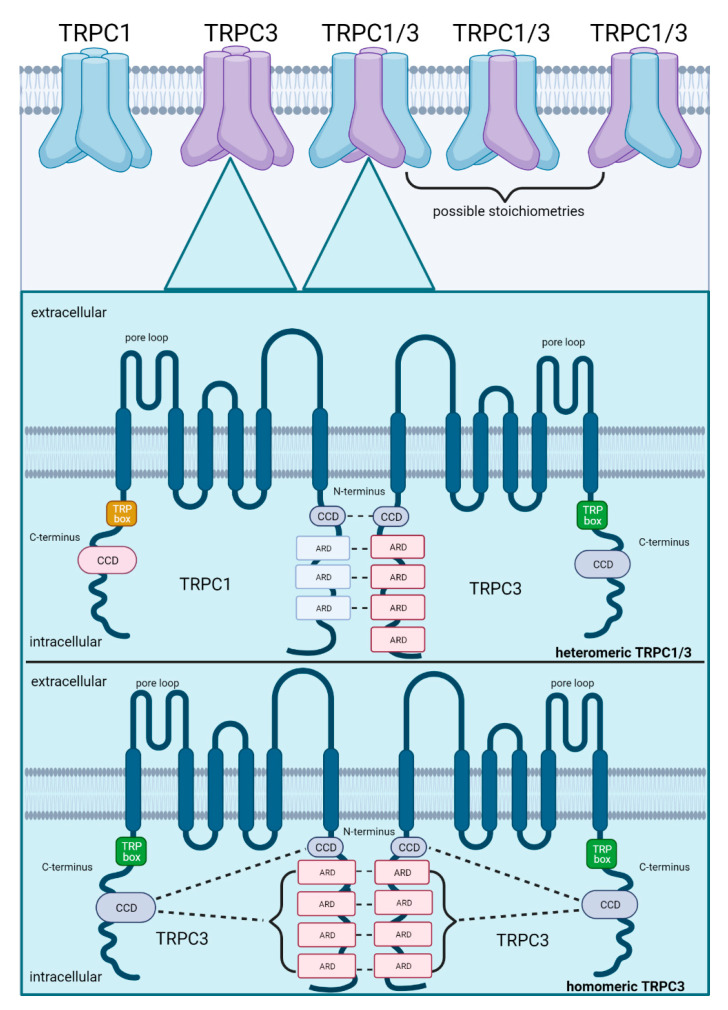
Schematic illustration of subunit assembly for heteromeric TRPC1/3 as compared to homomeric TRPC3. Co-expression of both subunits increases the probability of heteromerization, which is facilitated by their 28.7% amino acid congruency. Possible stoichiometries in a heteromeric assembly comprise 1:3, 2:2 or 3:1 (TRPC1:TRPC3). TRPC1/3 is stabilized by non-covalent interactions between ARDs (ankyrin repeat domains) and CCDs (coiled-coil domains) on their N-termini. In contrast, homomeric assembly of TRPC3 is stabilized by interactions between ARDs on their N-termini and by interactions between the CCDs on their C-termini with the ARDs and CCDs on their N-termini. Created with BioRender.com.

Thus far, the most important identified structural domains for stabilization of heteromeric TRP assemblies are ARDs and CCDs located on the N- and C-termini of the TRP subunits. To exemplify the molecular mechanisms that stabilize heteromeric assemblies, the well-described heteromeric TRPC1/3 is illustrated in Figure 2. Heteromeric TRPC1/3 is stabilized by non-covalent interactions between N-terminal ARDs and CCDs of the individual TRPC channel subunits whereas the homomeric TRPC3 assembly is stabilized by interactions between CCDs on the C-terminus with ARDS and CCDs on the N-terminus, indicating different molecular mechanisms for stabilization. Co-expression and heteromerization of TRPC1 and TRPC3 has been confirmed in skeletal myocytes [72]. In contrast to homomeric TRPC1 which promotes muscle regeneration after muscle atrophy [73], heteromeric TRPC1/3 plays an important role in the differentiation of myoblasts during myogenesis [72], consolidating the notion that heteromeric TRP assemblies are linked to other signal cascades than their homomers. The exact stoichiometry of TRPC1/3 heteromers (1:3, 2:2 or 3:1 for TRPC1:TRPC3, as shown in Figure 2) remains, however, unclear and may potentially vary with respective consequences for channel properties.

### 3.4. Properties of Heteromeric TRP Channel Assemblies

In line with different functional effects from homomeric channels, heteromeric assemblies of TRP channel subunits have a unique cation conductance that is unrelated to the additive conductance of the individual channel subunits. This was shown, for example, for the heteromeric assembly of TRPV1 and TRPV3 in HEK293 cells, which has an intermediate conductance compared to the homomeric assemblies of TRPV1 and TRPV3 [74]. Similarly, an intermediate conductance as compared to the homomeric assemblies was reported for TRPC1/P2 [21]. As such, TRP channels may adapt their individual conductivity (and putatively also their opening probability and their intracellular localization) by forming heteromers, thereby modulating cation influx and downstream cellular responses [21,29,62,75].

Interestingly, heteromeric TRP channels do not only show a unique cation conductance but also a distinct activation of downstream signaling pathways which is dependent on their cell type expression. Heteromeric TRPC1/3 as well as the heteromeric TRPC3/6/7 channel assembly have been reported to act as store-operated channels [73,76,77]. That notwithstanding, these assemblies activate different signaling pathways, as TRPC1/3-mediated Ca^2+^ influx plays a critical role in the differentiation of neuronal cells [73], whereas TRPC3/6/7 mediates an inflammatory response in astrocytes [62]. Heteromeric assemblies of TRP channel subunits have also been demonstrated in endothelial cells, e.g., TRPV4/C1/P2 in rat mesenteric artery endothelial cells (RMAECs) [64] and TRPV4/C1 in human umbilical vein endothelial cells (HUVECs) [7]. Heteromeric TRPV4/C1/P2 was shown to cause a flow-mediated Ca^2+^ influx, which is considered to play a major role in vasodilation, as it leads to the production of endothelial vasodilators like NO. A vasodilatory function in vascular endothelial cells has similarly been suggested for TRPV4/C1 which can be activated by stromal interaction molecule (STIM1) and Ca^2+^ release-activated calcium channel protein (Orai1), resulting in store-operated Ca^2+^ influx [7]. In addition to vasoregulation, maintenance and regulation of barrier properties is a basic function of pulmonary microvascular endothelial cells that is similarly regulated by TRP channel mediated Ca^2+^ influx. The resulting increase in intracellular Ca^2+^ concentration activates the calmodulin (CaM)/myosin light chain kinase (MLCK)-signaling pathway which in turn triggers cell contraction and additional Ca^2+^-dependent signaling cascades such as disassembly of intercellular junctions, ultimately resulting in increased endothelial permeability. Amongst others, TRPC1/4, TRPC3/6 and TRPV1/4 have been shown to be expressed and regulate barrier function in pulmonary microvascular endothelial cells. TRPC1/4 and TRPC3/6 present some of the best characterized heteromeric TRP channels, whereas TRPV1/4 is a recently discovered TRP heteromer. All three TRP heteromers will be described below in greater detail as they regulate barrier function and play an important role in lung inflammation.

## 4. Heteromeric TRPC1/4 Assembly

### 4.1. Heteromeric TRPC1/4 Structure

In pulmonary microvascular endothelial cells, TRPC1/4—just like TRPV4/C1 —interacts with Orai1, which in turn increases a store-operated Ca^2+^ influx through the heteromeric TRPC1/4 assembly [78]. The resulting Ca^2+^ influx via TRPC1/4 can increase pulmonary endothelial permeability and thus promote permeability-type lung edema in acute lung injury [1]. TRPC1 and TRPC4 share 41.3% of their primary structure and are therefore likely to co-assemble [20]. Typically, the quaternary structure of the canonical TRP subfamily is maintained by non-covalent interactions between the subunits’ C-termini and N-termini [79]. Specifically, for stabilization of the heteromeric TRPC1/4 assembly the CCD on the N-terminus and the structural domain from 725 to 745 on the C-terminus of TRPC1 interact with the CCD on the N-terminus and the structural domain from 673 to 725 on the C-terminus of TRPC4 [59].

### 4.2. TRPC1/4 Regulation

Regarding its activation mechanism, heteromeric TRPC1/4 assembly was not only found to be activated by Orai1 but also by a G_αq_-protein-coupled receptor in HEK293 whereby the G_αq_ subunit upon dissociation from the G-protein-coupled-receptor (GPCR) activates TRPC1/4 directly [80]. Additionally, Phospholipase Cβ (PLCβ) is activated by G_αq_-coupled GPCRs, inducing hydrolysis of phosphatidylinositol 4,5-bisphosphate (PI(4,5)P_2_), decreasing PI(4,5)P_2_ concentrations. The resulting decrease in PI(4,5)P_2_ levels reduces the activity of the TRPC1/4 channel, indicating that the activation of TRPC1/4 by the G_αq_-PLCβ pathway is self-limited [80]. Interestingly, TRPC1/4 was found to differ in its PI(4,5)P_2_ sensitivity [81] and to have a reduced Ca^2+^ conductance compared to homomeric TRPC4 [44], underlining that heteromerization changes the channel’s regulation and cation conductance. Although the Ca^2+^ conductance for TRPC1/4 is surprisingly lower compared to homomeric TRPC4, its mediated store-operated Ca^2+^ influx may be increased by prolonged channel opening that could be further extended by PI(4,5)P_2_ release which increases TRPC1/4-mediated Ca^2+^ influx [80].

### 4.3. TRPC1/4 Function

The resulting Ca^2+^ influx was shown to facilitate gap formation and thus increased endothelial permeability during acute pulmonary inflammation [1,82]. In contrast to that, TRPC1/4 is suggested to be protective in hypoxia-induced pulmonary arterial hypertension [83], where activation of nuclear factor-kappa B (NF-κB) and increased Galectin-3 expression shifts cellular response towards apoptosis by inactivation of TRPC1/4 and resulting suppression of autophagy [83]. TRPC1/4-mediated Ca^2+^ influx can on the one hand increase microvascular endothelial permeability and thus increase endothelial inflammation [1]. On the other hand, however, TRPC1/4-mediated Ca^2+^ influx has a protective effect in hypoxia-induced pulmonary arterial hypertension by maintaining autophagic responses [83], suggesting that the effect of TRPC1/4-mediated Ca^2+^ influx depends on the specific physiological or pathophysiological context, respectively.

## 5. Heteromeric TRPC3/6 Assembly

### 5.1. Heteromeric TRPC3/6 Structure

In human microvascular endothelial cells (HMVECs), TRPC3/6 can be activated by vascular endothelial growth factor (VEGF) and results in a Ca^2+^ influx [22]. The amino acid sequences of TRPC3 and TRPC6 show a 71.9% congruency and as such, of all TRP channel subunits they are the most probable pair to form a heteromer [20]. As mentioned before, the N-terminus of TRPC3 consists of four ARDs and a CCD whereas a CCD was also found on its C-terminus [54]. Heteromeric TRPC3/6 has been identified in FRET analyses, demonstrating that the 131 amino acids on the N-terminus, including the four ARDs, are crucial for stabilization of heteromeric TRPC3/6 assembly [28].

### 5.2. TRPC3/6 Regulation

TRPC3/6 is activated by intracellular diacylglycerol (DAG) [84] as a result of PI(4,5)P_2_ hydrolysis by PLC isoforms like PLCβ or PLCγ in response to, e.g., inflammatory stimuli [85]. In parallel with DAG, PI(4,5)P_2_ hydrolysis forms inositol 1,4,5-trisphosphate (IP_3_), inducing a store-operated Ca^2+^ entry (SOCE) through TRPC3/6 [76,86]. Whether TRPC3/6 acts as a store depletion- or GPCR-activated channel depends on its cell type-specific expression. In rat prostate smooth muscle cells [87], cardiac myocytes [88] and Jurkat T-cells [84], heteromeric TRPC3/6 has been shown to get activated by GPCR, whereas it shows a store-operated activity in mesangial cells [89]. Additionally, the TRPC3/6-mediated increase in intracellular Ca^2+^ concentrations can activate hypertrophic and anti-apoptotic signaling pathways. For example, brain-derived neurotrophic factor-mediated TRPC3/6 activation inhibited apoptosis in neonatal rat ventricular myocytes [90], whereas angiotensin II induced DAG release causes an activation of TRPC3/6 in cardiomyocytes, initiating a hypertrophic response [88].

### 5.3. TRPC3/6 Function

In pulmonary microvascular endothelial cells, TRPC3/6-mediated responses play a crucial role in acute pulmonary inflammation when VEGF is released and activates the heteromeric channel [91]. On the one hand TRPC3/6 activation promotes loss of endothelial barrier function in acute pulmonary inflammation [91]. TRPC3/6-mediated signaling might also be important to maintain endothelial barrier integrity on the other hand, as pro-angiogenic endothelial responses—mediated via VEGF and TRPC3/6—may counteract endothelial senescence and decrease permeability [92].

## 6. Heteromeric TRPV1/4 Assembly

### 6.1. TRPV1/4 Structure

Heteromeric TRPV1/4 assembly has been demonstrated by FRET analyses [74]. Yet, little is known so far about this heteromer. Typical for TRP heteromers, its cation conductance is dependent on its stoichiometry, with conductance closer resembling the homomeric TRPV1 assembly—characterized by very long openings and a low conductance in whole-patch clamp-recordings in HEK293 cells—with integration of more TRPV1 channel subunits into the heteromeric assembly [74]. Homomeric TRPV1 assemblies are known for their capsaicin-induced activation, which is dependent on an alanin residue in position 578 between the 5th and 6th span of the cation-permeable pore loop [93]. Interestingly, deletion of the amino acids 233 to 295 in the N-terminus reduces capsaicin-induced channel activity while FRET analyses demonstrated a weaker signal for TRPV1 tetramer formation, suggesting this amino acid, which includes the first two ARDs, as a possible stabilizing region for heteromerization with TRPV4. Additionally, the transmembrane domains have been proposed to stabilize the heteromeric assembly of TRPV1 and TRPV4, albeit this hypothesis remains to be proven [20].

### 6.2. TRPV1/4 Regulation

Both TRPV1 and TRPV4 channels can be activated by lipid-derived molecules like arachidonic acid [84,85] and by a variety of toxins (e.g., scorpion venom, botulinum neurotoxin and lipopolysaccharides) [94,95] and play important roles in mechano-, thermo-, osmo- and nociception depending on their cell type expression [67,96,97,98,99,100].

### 6.3. TRPV1/4 Function

In RMECs, TRPV1/4 was shown to regulate angiogenesis [27]. Heteromeric TRPV1/4 may also alter membrane potential similar to homomeric TRPV4-mediated Ca^2+^ influx which increases K^+^ efflux via activation of Ca^2+^-dependent K^+^ channels, such as intermediate and small Ca^2+^-activated K^+^ channels (IK and SK), causing hyperpolarization which in turn amplifies Ca^2+^ influx [101,102]. As such, TRPV1/4-mediated Ca^2+^ influx and endothelial hyperpolarization may stabilize each other in a positive feedback loop, thereby driving TRPV1/4 mediated endothelial responses such as angiogenesis but considering that angiogenesis does not take place in matured lungs.

## 7. Other Heteromeric TRP Assemblies

Heteromeric assembly of TRP channels thus emerges as a way for cells to combine and finetune individual properties of each channel to generate various channel complexes from the same genetic repertoire. As such, heteromeric assemblies may play an important role in regulating the Ca^2+^ responses to specific stimuli, in a cell- or tissue-specific manner, and/or as a function of the individual biochemical or biomechanical cellular environment. Over the past decades different endothelial and non-endothelial variations of heteromeric TRP channel complexes have been identified, e.g., TRP channel subunits TRPC1 and TRPC4 were found to heteromerize also with TRPC5 [28]. Heteromeric TRPC1/4/5 is considered as a GPCR-activated channel and has been reported to have a lower Ca^2+^ conductance but longer openings compared to homomeric TRPC4 and TRPC5 assemblies [103]. TRPC1/4/5 increases action potential-triggered excitatory postsynaptic currents in the hippocampus and has thus been proposed to play an important role in spatial working memory and flexible relearning [104]. Interestingly, heteromeric assembly has also been reported for TRPC1, TRPC4 and TRPC6 and to increase SOCE upon its upregulation in pulmonary arteries. Notably, this effect was not solely attributable to pulmonary arterial smooth muscle cells but also involved other pulmonary cells like endothelial cells, ultimately promoting pulmonary vascular remodeling in Sprague Dawley rats in response to smoking exposure [105].

Within the TRPV family, TRPV1/2, TRPV1/4 and TRPV5/6 are known heteromers of which the heteromerization of TRPV5 and TRPV6 is the best characterized but is most prominent in epithelia [17,28,74]. Both TRPV channels, TRPV5 and TRPV6, share 73.9% of their amino acid sequence [20]. In 2003, Hoendrop and co-workers first described a heteromeric assembly of both channels that was stabilized by N-glycosylation in the extracellular loop of a transmembrane domain [17]. One year later, FRET analyses by Hellwig and co-workers confirmed the heteromeric formation of TRPV5/6 in HEK293 cells [38]. In TRPV5/6 assemblies, five CaM binding sites have been identified [106]. TRPV5/6 regulates intracellular Ca^2+^ influx and becomes inactivated when Ca^2+^/CaM-complexes bind to their CaM binding sites [106], thus establishing a negative regulatory feedback loop. TRPV5 and TRPV6 have been proposed to play an important role in non-small lung cancer, based on the fact that their expression in lung tumor tissue of patient with non-small lung cancer is reduced. Patients with reduced expression of TRPV5 and TRPV6 had also a reduced median survival after surgical resection [107,108]. This suggests that dysregulated Ca^2+^ homeostasis due to loss of TRPV5 and TRPV6 may contribute relevantly to the malignancy of tumor cells. Different stoichiometric configurations in heteromerization of TRPV5/6 (e.g., 1:3, 2:2 or 3:1 for TRPV5:TRPV6) again give rise to different Ca^2+^ conductance of the heteromeric assembly thus fine-tuning cellular Ca^2+^ signaling [17]. Specifically, as the number of TRPV6 subunits in a heteromeric assembly of TRPV5/6 increases, the Ca^2+^-dependent inactivation accelerates [17]. We conclude that TRP heteromers play a crucial role in endothelial and non-endothelial cells by regulating cellular Ca^2+^ influx by modulating channel conductances and opening phases, thus regulating vascular inflammation and physiological functions.

## 8. Role of Heteromeric TRP Assemblies in Lung Inflammation

### 8.1. Introduction to Lung Inflammation

Barrier function and vasoregulation are primary functions of pulmonary microvascular endothelial cells and are impaired in acute pulmonary endothelial inflammation [109,110,111]. Frequently, this “dysfunction” of the pulmonary endothelium establishes a vicious circle as increased endothelial permeability will promote lung inflammation and vice versa. In this scenario, Ca^2+^ influx is a key mechanism of pulmonary endothelial permeability and as such, heteromeric TRP assemblies play an important role in endothelial inflammation.

### 8.2. Activation of Heteromeric TRPC1/4 and TRPC3/6 in Lung Inflammation

**Figure 3:**
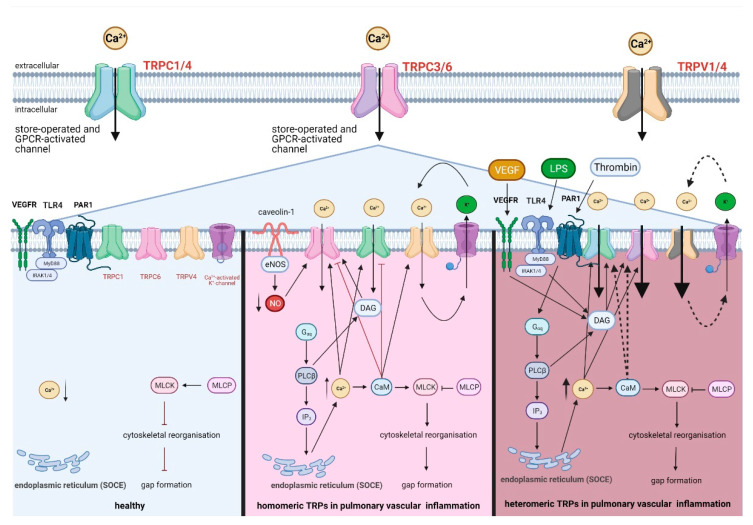
Regulation of endothelial permeability in acute pulmonary inflammation by homomeric and heteromeric TRP assemblies. Under physiological conditions (left), pro-inflammatory and barrier-disruptive receptors including TLR4 (Toll-like receptors), PAR1 (protease-activated receptor 1) and VEGFR (vascular endothelial growth factor receptor) are not activated by their respective ligands, and intracellular Ca^2+^ concentration is maintained low by closed confirmations of TRP homomers. In vascular inflammation with TRP homomers (centre), TRPC6 is activated by DAG (diacylglycerol) and by inhibition of NO production through eNOS (endothelial NO-synthase) uncoupling and eNOS interaction with caveolin-1. In parallel, TRPC1 is activated by IP_3_ (inositol 1,4,5-trisphosphate)-dependent Ca^2+^ release. TRPC6-mediated Ca^2+^ influx can then be inhibited in a Ca^2+^/CaM (calmodulin)-dependent manner as part of a negative feedback regulation which could (potentially) also be the case for TRPC1. TRPV4-mediated Ca^2+^ influx increases K^+^ efflux via intermediate and small Ca^2+^-activated K^+^ channels (IK and SK), causing endothelial hyperpolarization which in turn increases Ca^2+^ influx. In vascular inflammation with TRP heteromers (right), activation of PAR1, TLR4 and VEGFR by their respective ligands thrombin, lipopoly-saccharide (LPS) or VEGF, respectively, causes GPCR-operated activation of TRPC1/4 and TRPC3/6, and the resulting Ca^2+^ influx is stabilized by SOCE. The Ca^2+^ influx may be further stabilized via K^+^ efflux maintaining TRPV1/4 activation (dashed lines) and via Ca^2+^/CaM-complexes binding to their CaM binding sites in TRPC1/4 and TRPC3/6 (dashed lines). Heteromeric and homomeric TRP channel-mediated Ca^2+^ influx activates the CaM/MLCK (myosin light chain kinase)-signaling pathway by binding to CaM which in turn activates MLCK. Activated MLCK inhibits MLCP, thereby activating MLC II (myosin light chain II), actin-myosin interaction and formation of F-actin stress fibers. The resulting tensile force on endothelial cell–cell-junctions (inside-out-signaling) causes inter-endothelial gap formation and endothelial barrier failure. Created with BioRender.com.

To initiate an inflammatory response in specific, pathogens can be recognized by different endothelial receptors: Toll-like receptors (TLR), cytosolic NOD-like receptors (NLR), RIG-I-like receptors (RLR) and DNA sensors, which are commonly summarized as pathogen recognition receptors (PRR) [109]. Once activated, pathogen recognition receptors activate the NF-κB signaling pathway which in turn leads to the production of cytokines in pulmonary endothelial cells [111]. By releasing pro-inflammatory cytokines, monocytes and PMNs are recruited and start or aggravate the inflammatory response [109,111]. Among the PRRs, TLR4 is most important for recognizing lipopolysaccharides (LPS) from Gram-negative bacteria [112]. The activation of TLR4 increases the production of DAG, which in turn activates TRPC1/4 and TRPC3/6 [1,81,84] (Table 2), thus potentially further increasing Ca^2+^ influx via these assemblies in addition to their store-operated Ca^2+^ influx (Figure 3).

Additionally, thrombin activity is increased due to a dysregulation of coagulation in pulmonary inflammation [113]. Thrombin binds to and cleaves a specific GPCR, namely protease-activated receptor 1 (PAR1), leading to the secretion of VEGF and VEGFR-mediated signaling (Table 2), which in turn contributes to an increase of endothelial permeability [91,114] by DAG-dependent activation of heteromeric TRPC3/6 [84,115] as illustrated in Figure 3. PAR1 could also activate TRPC1/4 in pulmonary endothelial cells [116], as the G_αq_ subunit of PAR1 is released and initiates the formation of IP_3_ and thus, Ca^2+^ release from the endoplasmic reticulum (Figure 3). Heteromeric TRPC1/4 and TRPC3/6 could act in parallel and synergistically as GPCR- and Ca^2+^ store depletion-activated channels in acute pulmonary inflammation. The GPCR-induced Ca^2+^ influx via TRPC1/4 and TRPC3/6 heteromers may be further stabilized by their activation via Ca^2+^ store depletion from the endoplasmic reticulum [78,84,89], while TRPV1/4 mediated Ca^2+^ influx is amplified by Ca^2+^-dependent K^+^ efflux via IK and SK driving hyperpolarization. Both scenarios will ultimately result in prolonged and increased Ca^2+^ signaling in pulmonary microvascular endothelial cells, thereby promoting barrier loss and inflammatory signaling.

### 8.3. Activation of Homomeric TRPC1, TRPC6, TRPV1, and TRPV4 in Lung Inflammation

Platelet-activating factor is released during pulmonary inflammation and activates acid sphingomyelinase which produces ceramides, recruiting caveolin-1, endothelial NO-synthase (eNOS) and TRPC6 channels into caveolae [117]. Caveolar recruitment of caveolin-1 and eNOS inhibits NO production, increasing the activity of homomeric TRPC6 which in turn—via Ca^2+^ influx—activates the CaM/MLCK-signaling pathway [117]. When Ca^2+^/CaM-complexes bind to their CaM binding sites, TRPC6-mediated Ca^2+^ influx gets eventually inactivated, thereby limiting the endothelial response, which can be also applicable to TRPC1, under the assumption that TRPC1 itself induces a relevant Ca^2+^ influx and does not only modulate Ca^2+^ influxes through other Ca^2+^ permeable channels [118,119,120]. Other important homomeric TRP channels in lung inflammation are TRPV1 and TRPV4. TRPV1 contributes to ER-stress, is activated by high temperatures like fever in pulmonary microvascular endothelial cells [13] and in vascular inflammation in other vascular beds [121]. TRPV4 is a crucial regulator of lung vascular permeability [116]. Its Ca^2+^ influx is stabilized by hyperpolarization [101,102] and interestingly, by Ca^2+^/CaM-complexes binding to its CaM binding sites on its C-terminus [122]. This unveils a positive Ca^2+^/CaM-feedback loop for TRPV4 in contrast to TRPC1 and TRPC6. The Ca^2+^ influx stimulates the CaM/MLCK-signaling pathway, thus promoting endothelial barrier failure, e.g., in ventilator-induced lung injury in patients with the ARDS [14].

### 8.4. Comparison of Heteromeric TRP Assemblies to Homomeric TRP Assemblies in Lung Inflammation

TRPC1 and TRPC6 contribute to a self-limited Ca^2+^ influx whereas TRPV4 leads to an altered increased Ca^2+^ influx. An altered increased Ca^2+^ could be also assumed for TRP heteromers, as heteromers of TRPC1 and TRPC6, for example, are not reported to get inhibited by the negative Ca^2+^/CaM-feedback mechanism (Figure 3). One reason for that could be that CaM binding sites are covered in a heteromeric assembly and therefore not reachable for Ca^2+^/CaM-complexes. Another hypothesis could be that the binding of Ca^2+^/CaM-complexes to their binding sites has a positive effect on heteromers of TRPC1 and TRPC6, similarly to TRPV4. Additionally, a K^+^ efflux-mediated hyperpolarization caused by Ca^2+^ influx could increase the opening phase of TRPV1/4 just as it was found for TRPV4 (Figure 3). By loss of negative feedback loops and by activation through simultaneous upregulated activation mechanisms, TRP heteromers could have a longer opening phase. Therefore, TRP heteromers could induce an altered increased Ca^2+^ influx that promotes further endothelial loss of barrier integrity and thus would have a major pathophysiological relevance, e.g., in inflammatory processes by facilitating the invasion of immunological cells into the lung, such as monocytes, PMNs, T-helper 2 cells, mast cells and eosinophils.

## 9. Summary

Here, we discuss that TRP heteromers—similar to their homomeric TRP counterparts like TRPC1, TRPV4 or TRPC6 [4,14] —may play a crucial role in regulating endothelial barrier function. Heteromerization multiplies the variability of TRP channel compositions within their own subfamily and hence, of cellular responses to biochemical or biomechanical stimuli. Heteromeric TRP channels could also disinhibit reactions through loss of negative feedback loops. Therefore, TRP heteromers could be contributed a major, so far misunderstood, role in pulmonary vascular physiology and pathophysiology.

In pulmonary inflammation, the three here described TRP heteromers TRPC1/4, TRPC3/6 and TRPV1/4 have—to various extents—been shown to play a functional role in the regulation of barrier integrity. The knowledge of structural and functional advantages of TRP heteromerization remains to date largely elusive. Structural heteromeric compositions bind and stabilize via non-covalent interactions between ARDs or CCDs on their N- and C-termini [54,59] (Figure 2) and anchor in the plasma membrane by direct binding of PI(4,5)P_2_ to the C-terminus [124]. Functionally, TRPC1/4 and TRPC3/6 can be activated either by GPCR or Ca^2+^ store depletion from the endoplasmic reticulum [78,80,84]. As PLC-mediated PI(4,5)P_2_ hydrolysis from the phospholipid bilayer has a double-edged role on TRP heteromeric function in that it either diminishes channel activity by loss of PI(4,5)P_2_ relevant for anchoring of the heteromeric assembly to the plasma membrane and/or increases heteromeric channel activity via an increase in DAG [80,84]. In vascular inflammation, TRPC1/4 and TRPC3/6 may be simultaneously activated by GPCR and Ca^2+^ store depletion, with Ca^2+^ influx being initially triggered by GPCR signaling and subsequently stabilized by a SOCE mediated response.

The Ca^2+^ conductance in endothelial TRP heteromers has been found to be surprisingly intermediate compared to their corresponding homomeric assemblies [74]. As such, the question arises whether and how an intermediate Ca^2+^ conductance may increase endothelial permeability. A conceivable mechanism could be synergistic effects of an intermediate conductance with a prolonged opening by simultaneous activation in a GPCR- and Ca^2+^ store released-manner [7,73,78,125]. This might be very likely, as heteromeric TRP assemblies are activated during inflammation not only in pulmonary endothelial cells, but also in other cells [126].

For most heteromeric TRP assemblies the actual stoichiometry remains so far unclear. To obtain better in-depth insight into the structural makeup of TRP channel heteromeric assemblies and to foster our understanding in their physiological function, AFM or SC-SMD may be used for future identification of TRP heteromer’s stoichiometry in channel expressing cells. Both methods present relatively new approaches and allow for the analysis of heteromeric TRP channels at the single channel level, as they yield the three-dimensional structure of ion channels at the nanometer (for SC-SMD) or Ångström range (for AFM). With their high sensitivity, both methods represent promising alternatives to investigate the structure and the stoichiometry of heteromeric TRP channels.

Heteromeric TRP channel assemblies may amplify Ca^2+^ influx by prolonged openings, thus promoting a CaM/MLCK-signaling-dependent increase in endothelial permeability. Consistently, activation of heteromeric TRP channels can disrupt endothelial barrier function more profoundly as compared to homomeric TRP channels, and as such, more differentiated T-helper 2 cells, mast cells, neutrophils and eosinophils can invade lung tissues. In line with an important role of TRP channels in immune cell dynamics, TRPC6-positive cells correlated positively with the number of eosinophils in histological specimens from nasal tissue in patients with eosinophilic chronic rhinosinusitis with nasal polyps [127]. TRPC6 plays also an important role in allergic inflammation of the lung, as TRPC6 deficiency inhibited the immunological functions of T-helper 2, mast cells and eosinophils [128]. Analogously, heteromeric TRP channel assemblies containing TRPC6 may further amplify the invasion of eosinophils and therefore increase allergic inflammatory processes in the lung.

Heteromeric TRP assemblies may present possible therapeutic targets to reduce pulmonary vascular inflammation in diseases such as pneumonia, ARDS, COVID-19 or eosinophilic lung diseases.

## Figures and Tables

**Table 1:** Overview of methods for heteromeric TRP channels analyses.

Method	Application	Limitation	Example
AFM (atomic force microscopy) [36]	for stoichiometric determination in cells expressing tagged channels; allows for detection at the single channel level as it yields three dimensional structures in an Ångström (Å) range	not applicable for electrophysiological analysis	TRPP2/V4 [34]
Co-IP (co-immuno- precipitation) [37]	proof of interactions between TRP channel subunits in cells expressing tagged channels; allows for differentiation of heteromeric or homomeric assemblies	not applicable for structural and electrophysiological analysis	TRPV1/4 [38]
cross-linking in combination with mass spectrometry [39]	proof of interactions between TRP channel subunits and for structural and stoichiometric determination in native cells; provides a 3D-structure of interacting proteins, the distance between TRP channel subunits in the cross-linking reaction depends on the length of the crosslinker	not applicable for electrophysiological analysis	no applications made for heteromeric TRP channels yet
cryo-electron microscopy [40]	for structural analysis in cells overexpressing channels; allows for detection at the single channel level as it shows three dimensional structures, but sensitivity depends on the investigated protein’s electron irradiation and scattering	not applicable for electrophysiological analysis	no applications made for heteromeric TRP channels yet
FRET (Förster resonance energy transfer) [41]	proof of interactions between fluorescently tagged TRP channel subunits by live cell imaging; quantifies interaction over short distances (10–100 Å), yet fluorophores can alter binding properties	not applicable for structural, stoichiometric and electrophysiological analysis	TRPV1/4 [38]
molecular docking [31]	for prediction of structural domains that stabilize the TRP heteromer; method is based on acquired input data	not applicable for electrophysiological analysis	no applications made for heteromeric TRP channels yet
PLA (proximity ligation assay) [42]	proof of interactions between TRP channel subunits in native cells; quantifies interactions in a 30-nm range, allows quantification of heteromeric channel assemblies, but not at the single channel level; strongly depends on antibody specificity	not applicable for structural, stoichiometric and electrophysiological analysis	TRPV1/4 [27]
whole-cell patch-clamp recordings [43]	for electrophysiological analysis in native cells or cells expressing tagged channels; measures single ion channel current in a picoampere range	not applicable for structural and stoichiometric determinations	TRPC1/4 [44]
SC-SMD (single channel single molecule determinations) [45]	proof of interactions between TRP channel subunits and for structural and stoichiometric analysis in cells expressing tagged channels; detection at the single channel level as it yields three dimensional structures of proteins in a nanometer range	not applicable for electrophysiological analysis	no applications made for heteromeric TRP channels yet
x-ray crystallography [46]	for structural and stoichiometric analysis in cells overexpressing channels; detection at the single channel level as it yields three dimensional structures of molecules in an (Å) range, but is difficult to perform for transmembrane proteins	not applicable for electrophysiological analysis	no applications made for heteromeric TRP channels
Y2H (yeast two-hybrid technique) [47]	proof of interactions between TRP channel subunits in living native cells; detection of protein interactions by yeast reproduction, but not on a single channel level, difficult application for transmembrane proteins, and high rate of false positive results	not applicable for structural, stoichiometric and electrophysiological analysis	Drosophila retinal-specific TRP/L [29]

**Table 2:** An overview of activated receptors in lung inflammation and their link to TRP channels.

Receptor	Function in Lung Inflammation	Involved TRP Channels
TLR4 (Toll-like receptor)	activation of NF-κB signaling pathway and thus initiation of cytokine production; DAG production [111]	DAG production activates TRPC1/4, TRPC3/6, and TRPC6 [1,81,84]
PAR1 (protease-activated receptor 1)	secretion of VEGF and formation of IP_3_ [91,114]	activation of TRPC1/4 and TRPC3/6 via IP_3_-dependent Ca^2+^ release from the endoplasmic reticulum [84,115]
VEGFR (vascular endothelial growth factor receptor)	DAG production [115]	DAG production activates TRPC1/4, TRPC3/6, and TRPC6 [84,104,123]

## Abbreviations

AFM	atomic force microscopy
ARDS	Acute Respiratory Distress Syndrome
ARDs	ankyrin repeat domains
CaM	calmodulin
CCDs	coiled-coil domains
Co-IP	co-immunoprecipitation
DAG	diacylglycerol
eNOS	endothelial NO-synthase
FRET	Förster resonance energy transfer
GPCR	G-protein-coupled receptor
HEK293	human embryonic kidney cells 293
HMVECs	human microvascular endothelial cells
HUVECs	human umbilical vein endothelial cells
IK	intermediate conductance Ca^2+^-activated K^+^ channel
IP_3_	inositol 1,4,5-trisphosphate
LPS	lipopolysaccharide
RMAECs	rat mesenteric artery endothelial cells
MLCII	myosin light chain II
MLCK	myosin light chain kinase
MLCP	myosin light chain phosphatase
MRLC	myosin II regulatory light chains
NF-κB	nuclear factor-kappa B
Orai1	Ca^2+^ release-activated calcium channel protein
PAR1	protease-activated receptor 1
PI(4,5)P_2_	phosphatidylinositol 4,5-bisphosphate
PLA	proximity ligation assay
PLCβ/γ	phospholipase C β/γ
PMNs	polymorphonuclear leukocytes
PRR	pathogen recognition receptors
RMECs	retinal microvascular endothelial cells
ROS	reactive oxygen species
SC-SMD	single channel single molecule detection
SK	small Ca^2+^-dependent K^+^ channel
SOCE	store-operated Ca^2+^ entry
STIM1	stromal interaction molecule
TIRF	total internal reflection fluorescence microscopy
TLR	Toll-like receptors
TRP	transient receptor potential
VEGF	vascular endothelial growth factor
Y2H	yeast two-hybrid
